# Neuroscience-based nomenclature (NbN): the Portuguese version of the new classification for psychopharmacological drugs

**DOI:** 10.47626/2237-6089-2023-0771

**Published:** 2025-04-17

**Authors:** José Carlos Fernandes Galduróz, Aleksander Roberto Zampronio, Maria Aparecida Barbato Frazão Vital, Paulo Clemente Sallet, Joseph Zohar, Roberto Andreatini

**Affiliations:** 1 Universidade Federal de São Paulo Departamento de Psicobiologia São Paulo SP Brazil Departamento de Psicobiologia, Universidade Federal de São Paulo, São Paulo, SP, Brazil.; 2 Universidade Federal do Paraná Departamento de Farmacologia Curitiba PR Brazil Departamento de Farmacologia, Universidade Federal do Paraná, Curitiba, PR, Brazil.; 3 Universidade de São Paulo Faculdade de Medicina Departamento de Psiquiatria São Paulo SP Brazil Departamento de Psiquiatria, Faculdade de Medicina, Universidade de São Paulo, São Paulo, SP, Brazil.; 4 Tel Aviv University Post-Trauma Center Sheba Medical Center Tel Aviv Israel Post-Trauma Center, Sheba Medical Center, Tel Aviv University, Tel Aviv, Israel.

**Keywords:** Neuroscience, nomenclature, psychiatry, psychopharmacology, rational prescription

## Abstract

Neuroscience-based nomenclature (NbN) is a proposal to provide a nomenclature based on neuroscience and pharmacology instead of the old disease-based classification. The NbN is based on the mechanism of action and pharmacological target and aims to assist in rational prescription, to reduce stigma, and to increase treatment adherence. Currently, the NbN is endorsed by many psychiatric associations, has been adopted by several relevant journals, and is included in major psychiatry textbooks. Therefore, it is important that the NbN is known to psychiatrists.

The discovery of chlorpromazine as a medication able of providing sedation without altering the level of consciousness inaugurated the so-called psychopharmacological revolution.^1^ Since then, many other molecules have been synthesized and classified according to their clinical indications, such as antidepressants, anxiolytics, antipsychotics, mood stabilizers, and hypnotics.

Since the 1960s, the World Health Organization (WHO) has been concerned with establishing a globally accepted classification system to standardize studies on drug utilization. Thus, the Drug Utilization Research Group (DURG) was created, which developed the Anatomical Therapeutic Chemical (ATC) classification, based on the organ or physiological system on which each substance acts and its therapeutic properties and chemicals.^2^ However, this classification does not reflect either the advances in neuroscience that have taken place in the last 40 years or the current clinical indications. For example, fluoxetine is classified as an antidepressant, but its prescription goes much further, as it is prescribed for the treatment of bulimia nervosa, obsessive-compulsive disorder, panic disorder, posttraumatic stress disorder (PTSD), social phobia, and premenstrual dysphoric disorder. Other examples include lamotrigine, originally an anticonvulsant, but currently also prescribed as a mood stabilizer and dopamine blockers ("antipsychotics") like amisulpride and aripiprazole that are also used (in small doses) as augmentation agents in depression. There are no psychopharmacological drugs that have only a single indication, reflecting the complexity of the pathophysiology and treatment of mental disorders.

Currently, good clinical practice recommends sharing the proposed treatment guidelines with the patient, since psychoeducation is an important aspect of the treatment process. Explaining the pathology and the recommended prescription appear to increase patient adherence to treatment. It is not surprising that the current nomenclature is confusing for patients, as we often prescribe "antidepressants" for anxiety disorders or "antipsychotics" for depressed patients who do not have evidence of psychosis. Therefore, a new nomenclature is needed that encompasses current knowledge related to the pharmacology of drugs and their mechanisms of action, relating them to appropriate clinical indications.

The proposition of a new nomenclature was implemented by a task force that included five major international scientific neuropsychopharmacology organizations, the European College of Neuropsychopharmacology (ECNP), the American College of Neuropsychopharmacology (ACNP), the Asian College of Neuropsychopharmacology (AsCNP), the International College of Neuropsychopharmacology (CINP), and the International Union of Basic and Clinical Pharmacology (IUPHAR).^3^ This task force created the neuroscience-based nomenclature (NbN), aiming to provide a neuroscience and pharmacology-based (rather than disease-based) nomenclature that incorporates understanding of how psychopharmacological drugs work. In addition, the NbN should also decrease the stigma of treatment with these agents, increasing adherence through a nomenclature system that establishes the justification for selection of a specific drug.

The NbN classification is based on two main axes: pharmacology (pharmacological target) and mechanism of action (type of action on the target). Four additional dimensions are also included: (a) approved indication (approved by regulatory agencies such as the Food and Drug Administration [FDA] and European Medicine Agency [EMA]); (b) efficacy (indications not formally approved by the agencies but that have authoritative evidence of efficacy) and side effects; (c) practical notes; and (d) neurobiology. It is important to stress that our current knowledge about the mechanism of action and pharmacology of many drugs is flawed or incomplete, but describing this contemporary knowledge is better for clinical practice than waiting for a more definitive conclusion.^4^

Currently, 146 psychopharmacological agents are included in the NbN, accessible via a mobile app that can be freely downloaded from Google Play and the Apple Store. Following the idea that the NbN will be a common system shared by clinicians and researchers throughout the world, several important journals have adopted it as the nomenclature for citing psychopharmacological drugs in their articles (e.g., Biological Psychiatry, European Neuropsychopharmacology, and the International Journal of Neuropsychopharmacology), and it has been included in relevant books on psychiatry (e.g., the next edition of Sadock's Comprehensive Textbook of Psychiatry, Tasman's Textbook of Psychiatry, the Oxford Textbook of Psychiatry, and Stahl's Essential Psychopharmacology). The NbN is also endorsed by many psychiatric societies, such as the American Psychiatric Association (APA), the European Psychiatric Association (EPA), the German Association of Psychiatry, Psychotherapy and Psychosomatics (DGPPN), the Spanish Society of Psychiatry and Mental Health (SEPSM), and the Japanese Society of Psychiatry and Neurology (JSPN). Moreover, the NbN app has been translated and adapted to French, Spanish, and Japanese.

Using the NbN app, clinicians can search for the name of the medicine (generic or branded) to access information about its pharmacology, mode of action, approved indications, effectiveness, and side effects. Moreover, the former nomenclature is also available. An example of the NbN app info about alprazolam can be observed in Table 1. Different search strategies are also possible (e.g., by the mechanism of action or former terminology). It is worth mentioning that this application is being constantly updated and suggestions from its users for the inclusion of new drugs are welcome.

**Table 1:** Information given by the NbN app (English version) when "alprazolam" is typed

NbN classification		
Pharmacological target		GABA
Mechanism of action		PAM
Approved indications		Generalized anxiety disorder, panic disorder, short-term treatment of anxiety, alcohol withdrawal (France)
Efficacy		Anxiolytic, muscle relaxant, anticonvulsant, sleep promoter
Adverse effects		Sedation, drowsiness, ataxia, muscle relaxation, memory deficit
Committee notes		Many different benzodiazepine receptor agonists are licensed worldwide, some in most countries and some in only or two. They all act in the same way, and are distinguished only by pharmacokinetics unless otherwise indicated. Half-life 8-15 hours and it is metabolized by the cytochrome 3A4. Inhibitors of 3A4, like fluoxetine, erythromycin, ketoconazole, but also oral contraceptives, reduce its clearance. The herb kava will robustly reduce its clearance, whereas St John's wort will increase it. Synergistic effects with alcohol can produce severe sedation, behavioral changes, and intoxication.
Additional data		
	Former terminology	Anxiolytic
	Neurobiology	Pharmacology and mode of action: GABA positive allosteric modulator, GABA-A receptor, benzodiazepine site
		Neurotransmitter effects preclinical: Binds to GABA-A receptors
		Neurotransmitter effects human: Non-selective PAM
		Physiological preclinical: Reduces motor activity, conflict behavior, and promotes sleep, anti-epilepsy
		Physiological human: Increases fast activity on EEG, myorelaxant, anxiolytic, sedating, slows eye saccades, promotes sleep
		Brain circuits human: Broad action across all brain regions
	Pregnancy	D - positive evidence of risk
	Brand names	Xanax, alprazolam intensol, niravam

EEG = electroencephalogram; GABA = gamma-aminobutyric acid; NbN = neuroscience-based nomenclature; PAM = positive allosteric modulator.

Recently, Dr Joseph Zohar (NbN task force coordinator and current president of the CINP) contacted us to compose a Brazilian committee for the translation of NbN to Brazilian Portuguese, adapting it to medicines available commercially in Brazil. This Portuguese version of the NbN 3rd edition was launched recently (https://nbn2r.com/).

Finally, a new nomenclature is not sufficient to change the current picture and, thus, it is fundamental that the NbN be publicized and taught to new psychiatrists and clinicians to achieve its goals.^5^ We think that the Portuguese version of NbN will help in this task in Brazil.
